# Accurate Indoor Localization Based on CSI and Visibility Graph

**DOI:** 10.3390/s18082549

**Published:** 2018-08-03

**Authors:** Zhefu Wu, Lei Jiang, Zhuangzhuang Jiang, Bin Chen, Kai Liu, Qi Xuan, Yun Xiang

**Affiliations:** 1 College of Information Engineering, Zhejiang University of Technology, Hangzhou 310023, China; wuzf@zjut.edu.cn (Z.W.); 2111603003@zjut.edu.cn (L.J.); 201403090110@zjut.edu.cn (Z.J.); lk@zjut.edu.cn (K.L.); xuanqi@zjut.edu.cn (Q.X.); 2 School of Design, Zhejiang University of Technology, Hangzhou 310023, China; cjb@zjut.edu.cn

**Keywords:** indoor localization, CSI, visibility graph

## Abstract

Passive indoor localization techniques can have many important applications. They are nonintrusive and do not require users carrying measuring devices. Therefore, indoor localization techniques are widely used in many critical areas, such as security, logistics, healthcare, etc. However, because of the unpredictable indoor environment dynamics, the existing nonintrusive indoor localization techniques can be quite inaccurate, which greatly limits their real-world applications. To address those problems, in this work, we develop a channel state information (CSI) based indoor localization technique. Unlike the existing methods, we employ both the intra-subcarrier statistics features and the inter-subcarrier network features. Specifically, we make the following contributions: (1) we design a novel passive indoor localization algorithm which combines the statistics and network features; (2) we modify the visibility graph (VG) technique to build complex networks for the indoor localization applications; and (3) we demonstrate the effectiveness of our technique using real-world deployments. The experimental results show that our technique can achieve about 96% accuracy on average and is more than 9% better than the state-of-the-art techniques.

## 1. Introduction

Indoor localization is an important technique and can be used in numerous real-world applications [1,2,3]. However, unlike outdoor positioning, indoor localization remains a very challenging and largely unsolved problem. Being able to accurately determine indoor personal positions is a nontrivial task. For example, the indoor environment can be complex, unpredictable, and mingled with walking personals. Therefore, in this work, we propose a novel and accurate indoor localization technique which combines the statistics based and network based CSI features.

Indoor localization is widely used in many wireless applications. The localization accuracy and efficiency demand is ever increasing. The specific application scenarios of indoor location techniques include object tracking, personnel localization, elderly care, somatosensory games [4], etc. Under certain emergent conditions, such as fire rescue, an accurate, efficient and real-time localization scheme can possibly save many lives. However, traditional indoor location techniques employ a received signal strength indicator (RSSI) as the reference physical indicator to determine locations [5,6]. The main problem of RSSI is its lack of stability and sensitivity. It can easily fluctuate over time, resulting in low localization accuracy. Therefore, we use the more stable and accurate CSI as the reference physical indicator [7,8,9].

However, there are still many challenges for the existing CSI based indoor localization methods. One major problem is that in their models they typically do not consider the inter-subcarrier correlations. Most existing algorithms only utilize the amplitude and phase information of individual subcarriers. However, the frequency correlations among neighboring subcarriers can also contain important location related information. Therefore, we propose using the VG method [10] to model the inter-subcarrier correlations. The VG technique can transform time or frequency series data into a complex network. The connections of the network nodes are determined by the geometric visual relations of adjacent sequential points. Through the construction of the corresponding complex network, we can derive a CSI based network with subcarriers as the network nodes and the geometric visibility as the edges. It can reflect the frequency correlations between adjacent subcarriers. Based on the network, we extract corresponding network features for indoor localization.

To improve the localization accuracy, we propose a passive indoor location technique based on both statistical and network CSI features. Our technique employs the fingerprint library and can be divided into training and testing stages. During the offline training stage, the CSI information is collected and pre-processed. Then, we construct the VG based complex network from the collected 30 sequential subcarriers. After that, both the network and statistical features are extracted and processed by the appropriate machine learning techniques to form the fingerprint library. During the online testing stage, the testing data are processed similarly. Then, machine learning algorithms are used to classify and estimate indoor locations.

In general, in this work, we have made the following main contributions:

1. We propose a novel passive indoor localization algorithm, which combines both intra-subcarrier statistics features and inter-subcarrier network features. It can greatly improve the localization performance.
2. We develop a modified VG based method to process the frequency-series subcarrier data. Our approach can explore the intra-subcarrier and inter-subcarrier correlations between adjacent subcarriers.
3. We validate our theories and techniques in real-world deployments. The results confirm that our technique can significantly outperform state-of-the-art indoor localization techniques and are more robust as well.

The remaining parts of this article are organized as follows: Section 2 describes the related work; Section 3 introduces the relevant theoretical knowledge; Section 4 presents our technique; Section 5 discusses the experimental setup and the analysis of the results; Section 6 summarizes the full text.

## 2. Related Work

The related work can be generalized into two categories, which are indoor localization techniques and complex network techniques, respectively.

### 2.1. Indoor Localization Techniques

Indoor localization is important for many mobile applications. Therefore, accurate and effective indoor positioning has received widespread research interests. Previously, wireless indoor localization techniques mainly rely upon RSSI [11,12,13,14], which can be inaccurate, unstable, and vulnerable to multi-path effects. Therefore, CSI based techniques are becoming mainstream. Sen et al. [15,16] propose PinLoc, which is based on the CSI fingerprint library. It can achieve meter level localization accuracy. Wu et al. develop FILA, a fine-grained indoor localization technique [17]. Shaw et al. design the Pilot algorithm [18]. Nasser et al. propose the MonoPHY technique [19]. Wu et al. propose a passive indoor localization technique based on CSI and Naive Bayes classifier [20]. There are problems and challenges for the existing CSI based localization techniques. The traditional CSI based localization methods only utilize the statistical features extracted from the individual subcarrier and do not consider the relationship between subcarriers. However, the relationship may contain important position information. Thus, it is important to quantify the relationship of adjacent subcarriers.

### 2.2. Complex Network Techniques

Complex network refers to a network with the properties of self organization, self similarity, attractor, small world, and scale free degrees [21]. In real-world applications, many complex systems, such as power grids, aeronautical networks, traffic networks, internet, and social networks, etc., can be modeled using complex networks [22,23,24]. Meanwhile, the visibility graph is an efficient and thus widely used technique to construct complex networks from complex systems, e.g., CSI. Lacasa et al. proposed the VG based technique to transform time series data into complex networks [10]. Gao et al. improve upon the original VG method and extract the network features for epileptic classification [25,26]. Yan et al. utilize the VG technique to extract network features and distinguish traffic states [27]. Zhu et al. employ multiple VG techniques to transform human sleep data into complex networks and extract network features for sleep classification [28]. Thus, existing research works demonstrate that using VG network to transform time series data into complex networks can reveal the internal relationship of data and improve classification results. Therefore, we propose to transform the time series CSI data into complex network.

## 3. Preliminaries

In this section, we describe the basic theories and properties of CSI and VG.

### 3.1. CSI and Localization

Compared with RSSI, CSI can provide more detailed and fine-grain subcarrier information. The channel frequency response (CFR) of a typical wireless channel can be expressed using the following equation:

(1) Y=HX+N,

where *X* is the transmitting signal vector, *Y* is the receiving signal vector, *H* is the channel state matrix and *N* is the Gaussian white noises. Thus, the channel state *H* can be calculated using the following equation:

(2) $$\hat{H}=\frac{Y}{X},$$

where $\hat{H}$ is the CFR in the frequency domain. The corresponding CSI information can be extracted from a WiFi wireless adapter. For subcarrier *k* with central frequency $f_{k}$, its CSI is defined as $H(f_{k})$. The CSI signature contains both amplitude and phase information, which is defined in the following equation:

(3) $$H\left(a\right)=\left|H\left(a\right)\right|e^{jsin∠H(a)},$$

where H(a) is the CSI for the ath subcarrier, |H(a)| is its amplitude, and ∠H(a) is its phase. For multiple in multiple out (MIMO) systems with multiple receiving and transmitting antennas, CSI of each subcarrier can be expressed as a p×q matrix, where *p* is the number of transmitting antennas and *q* is the number of receiving antennas. Thus, for each data packet, we can derive a p×q×N matrix, where *N* is the total number of subcarriers. In this work, the number of *N* is set to 30 [29].

**Figure 1:**
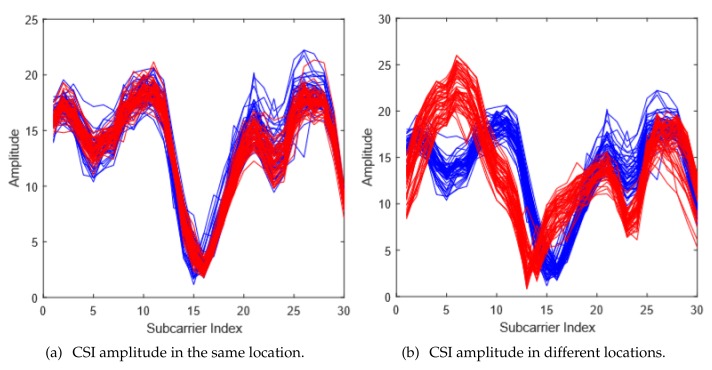
The CSI amplitude comparison.

The underlying reasoning for applying the CSI information in indoor localization applications is that, when the personnel are in different locations, the signal paths and the corresponding multi-path effects vary. Thus, we can observe different reactions and variations on the CSIs. To better identify the human location, the CSI signature should be both stable in the same location and differentiable in the different locations. This characteristics can be readily observed in Figure 1.

### 3.2. VG Introduction

**Figure 2:**
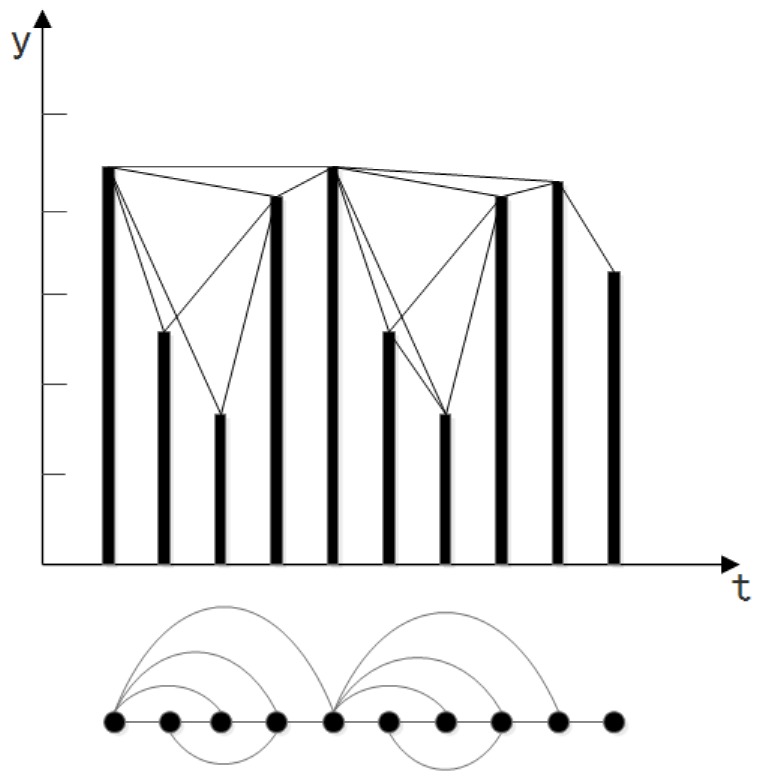
A VG construction example for time series data.

The VG method is widely used to transform time series data into complex networks [10]. Assuming there are three different points in the time series data space, which are denoted as *a*, *b*, and *c*. The network can be constructed using the following equation:

(4) $$y_{c}<y_{b}+\left(y_{a}-y_{b}\right)\frac{t_{b}-t_{c}}{t_{b}-t_{a}},$$

where *y* represents the value on the *y*-axis and *t* represents time. Thus, based on Equation (4), we can derive an undirected complex network. A complex network construction example is shown in Figure 2.

### 3.3. Machine Learning Algorithms

We introduce several widely used machine learning algorithms and implement them later.

#### 3.3.1. Bayesian Network

Bayesian network (BNet) models the process of human cognitive reasoning. It utilizes a directed acyclic graph (DAG) and its associated conditional probability tables to model the causal inference relation of uncertain events. Each node in DAG represents a random variable, which can be directly observed or hidden. Edges represent the conditional dependency between corresponding variables. Each element in the conditional probability table represents the joint conditional probability between the node and its precedents. By training the Bayesian network, the conditional probability table can be filled and thus used for classification.

#### 3.3.2. Support Vector Machine

Support vector machine (SVM) is a powerful and widely used machine learning technique. The key is to find the boundary hyper plane among different categories, so that different types of samples can be as far away from the boundary hyper plane as possible. Thus, SVM can exhibit an excellent classification ability. Recent SVM techniques utilize various kernel functions, e.g., Gaussian kernel function, to project the plane into a curved surface and greatly improve the performance and application scenarios. SVM is typically resilient to attacks and noises.

#### 3.3.3. Random Forest

Random forest (RF) is an ensemble machine learning algorithm for tasks like classification and regression, etc. Its weak classifiers typically use the classification and regression tree. It first generates a large quantity of decision trees by random selection, and then combines the results of these decision trees to make the final classification decision. Random forest is widely used in real-world applications. Since RF utilizes random sampling, it can minimize modeling variance and have outstanding generalization ability. Therefore, RF is usually resilient to attacks and accurate in general.

## 4. VG Based Indoor Localization Method

**Figure 3:**
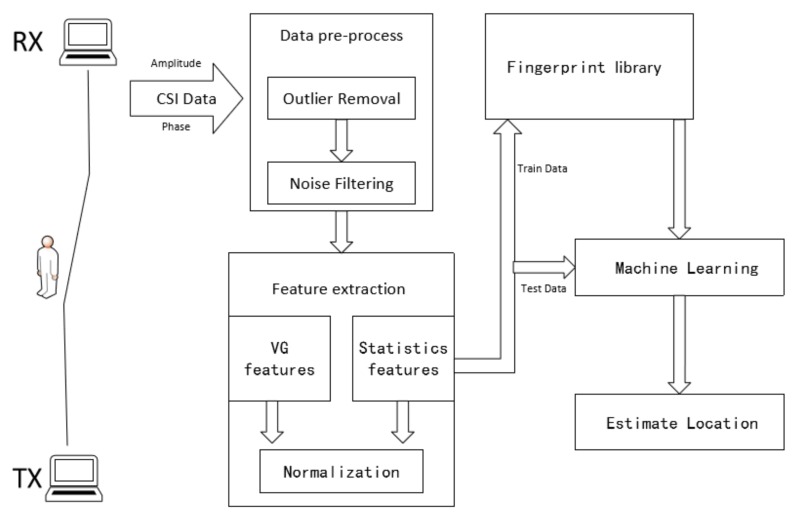
The system flowchart.

Our proposed technique includes three parts, which are VG network construction, network feature extraction, and CSI fingerprint library. The flow of our method is presented in Figure 3.

### 4.1. VG Network Construction

The CSI signatures can be considered as frequency series data. Thus, to explore the correlations between adjacent subcarriers, we propose using the VG technique to transfer frequency series CSI signatures to complex networks. The CSI data contain both amplitude and phase information. Specifically, assuming that there are three different subcarriers *a*, *b*, and *c*, we use the following equation to derive the VG network for amplitude:

(5) $$A_{c}<A_{b}+\left(A_{a}-A_{b}\right)\frac{f_{b}-f_{c}}{f_{b}-f_{a}},$$

where, for the ith subcarrier, $A_{i}$ represents its amplitude and $f_{i}$ represents its frequency.

Similarly, the CSI phase information can also be used to construct the corresponding VG network using the following equation:

(6) $$P_{c}<P_{b}+\left(P_{a}-P_{b}\right)\frac{f_{b}-f_{c}}{f_{b}-f_{a}},$$

where $P_{i}$ is the phase value for the ith subcarrier.

**Figure 4:**
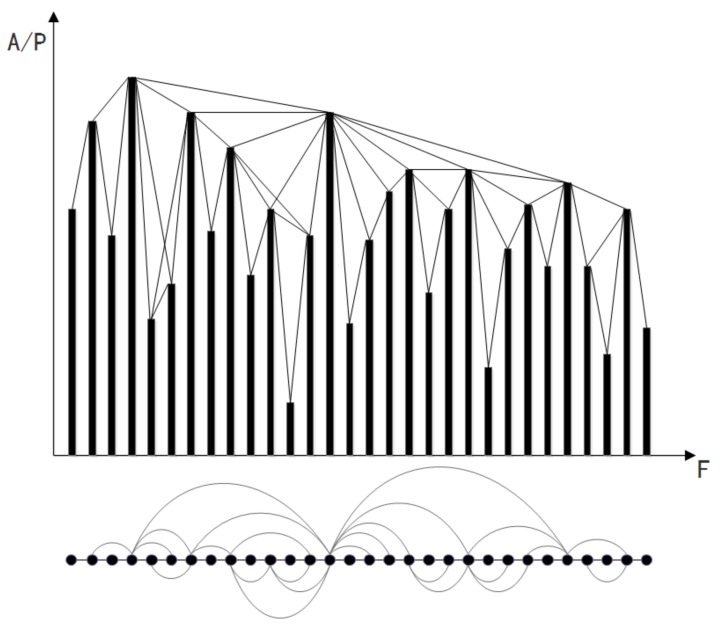
The VG construction process for frequency series CSI data.

Figure 4 shows an example of transforming 30 subcarriers into the VG based complex network. In the figure, the *x*-axis is the subcarrier frequencies and the *y*-axis is the amplitude. By applying the VG based network construction rule as presented in Equation (5), we can derive the corresponding complex network.

In general, we construct an undirected and acyclic network from frequency series CSI signature. We first assign subcarrier indices as network nodes. Then, we apply the VG technique to establish the connections between nodes. Thus, we can use existing network techniques to analyze the CSI data and greatly increasing the number of features for classification.

### 4.2. Network Feature Extraction

After the construction of CSI complex networks, we extract the network features. In this work, we utilize the following features:

1. Degree deviation [25]
  The degree deviation can be calculated using the following equation:
  (7) $$\begin{array}{c} k_{std}={\left(\frac{\sum_{i=1}^{N}{\left(k_{i}-\bar{k}\right)}^{2}}{N-1}\right)}^{\frac{1}{2}},\\ \bar{k}=\frac{1}{N}\sum_{i=1}^{N}k_{i}, \end{array}$$
  where $k_{i}$ represents the number of nodes connecting to node *i*, $k_{std}$ represents the degree deviation, $\bar{k}$ represents the average degree, and *N* is the total number of nodes in the network.
2. Degree assortativity coefficient [30]. The Degree assortativity coefficient feature can be extracted using the following equation:
  (8) $$r=\frac{M^{-1}\sum_{i}j_{i}k_{i}-{\left[M^{-1}\sum_{i}\frac{1}{2}\left(j_{i}+k_{i}\right)\right]}^{2}}{M^{-1}\sum_{i}\frac{1}{2}\left(j_{i}^{2}+k_{i}^{2}\right)-{\left[M^{-1}\sum_{i}\frac{1}{2}\left(j_{i}+k_{i}\right)\right]}^{2}},$$
  where $j_{i}$ and $k_{i}$ is the degree of the two nodes connected by edge *i* and *M* is the total number of edges.
3. Clustering coefficient entropy [25] The clustering coefficient entropy feature is extracted as follows:
  (9) $$\begin{array}{c} C_{i}=\frac{\tau_{i,\Delta }}{\tau_{i}};\;\;\;P_{c,i}=\frac{C_{i}}{\sum_{i=1}^{N}C_{i}},\\ Ec=-\sum_{i=1}^{N}\left(P_{C,i}\right)log\left(P_{C,i}\right), \end{array}$$
  where, for node *i*, $C_{i}$ is its local clustering coefficient, $\tau_{i,\Delta }$ is the total number of edges connecting to all the neighboring nodes of *i*, $\tau_{i}$ is the the number of edges connecting to node *i*, $P_{C,i}$ is the clustering coefficient probability, *N* is the total number of nodes in the network, and $E_{C}$ is the clustering coefficient entropy.
4. Average weighted degree [31]. The average weighted degree feature can be extracted as follows:
  (10) $$w_{ab}=\frac{1}{M}\sum \left[arctan\frac{y\left(x_{b}\right)-y_{x_{a}}}{x_{b}-x_{a}}\right],$$
  where *a* and *b* are two separate nodes, *x* is the corresponding subcarrier frequency, *y* is amplitude or phase, and *M* is the total number of edges.

### 4.3. Fingerprint Library Creation

#### 4.3.1. Statistical Feature Extraction

The raw data of 30 subcarriers contain large amounts of noises and useless redundant signals and, thus, usually cannot be used for classification directly. Otherwise, they can easily cause overfitting and lead to inferior classification performance. Thus, it is common practice to generalize the statistical features from the raw data. In this work, we calculate and utilize four statistical features, which are standard deviation (STD), median absolute deviation (MAD), mean value (MEAN), and median value (MEDIAN), respectively. We do not include the maximal and minimal traits in this work. Experimental results show that, by adding those two features, the classifiers become more vulnerable to overfitting and tend to derive deteriorated results.

#### 4.3.2. Fingerprint Library

There are significant variations in derived feature magnitudes. Therefore, we normalize the features using the Z-score method as follows:

(11) $$F_{norm}=\frac{F-\mu }{\sigma },$$

where $F_{norm}$ is the normalized features, *F* is the original features, μ is the mean value, and σ is the standard deviation. The normalized features, including both statistical and network ones, are combined to create the final fingerprint library.

## 5. Experimental Results

### 5.1. Experiment Setup

The experiment platform includes two parts, which are access point (AP) and monitoring point (MP), respectively. The CSI signature is extracted using a CSI-tool [29]. For the experiment, we deploy two notebook equipped with Ubuntu 14.04 (Canonical, London, UK) and Intel 5300 network adapter (Samsung, Suzhou, China). The adapter has three MIMO antennas and thus can form six link pairs in theory. However, in the real-world environment, we can only extract three link pairs stably. Thus, we choose link pair 1-1, 1-2, and 1-3 in this work. The machine learning algorithms are run on a ASUS FH5900V computer (Shanghai, China) equipped with Intel i7-6700HQ CPU and 8 G memory.

**Figure 5:**
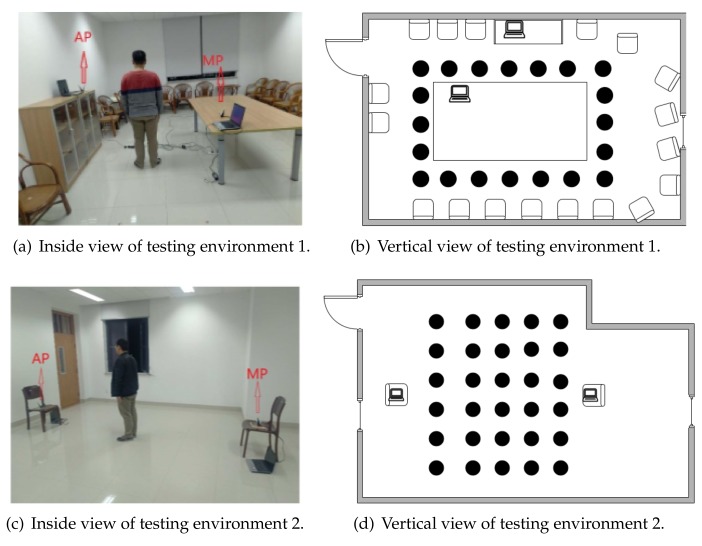
The experiment environments.

During the experiment, the testing participator is standing on the different locations in the room. The AP sends 100 data packets per second and lasts for 10 s. Therefore, at each location, we collect 1000 samples. There are two different testing environments as shown in Figure 5. Figure 5a shows the inside view of testing environment 1, which is a small and noisy conference room. Figure 5b is its vertical view and the room size is approximately $5\times 6\;m^{2}$. Figure 5c shows the inside view of testing environment 2, which is an empty and quiet class room. Figure 5b is its vertical view and the room size is approximately $6\times 8\;m^{2}$.

For both environments, the distance between each data collection point is around 1 m, i.e., the localization resolution is set to be 1 m. In testing environment 1, the MP is placed at 1.2 m high, while the AP is placed at 0.5 m high. Their distance is 2.5 m. In testing environment 2, both AP and MP are placed at 0.5 m high with a distance of 7 m. At each location, we randomly select 600 samples as the training set, and the rest form the testing set.

### 5.2. Data Pre-Processing

The data pre-processing includes amplitude and phase extraction, abnormality processing, and data smoothing.

#### 5.2.1. Amplitude and Phase Extraction

The CSI amplitude information is stable and easy to extract. Thus, it is the most widely used feature in indoor localization applications. However, the phase information contains significant noise and synchronization problems. Thus, the original phase information must be linearized [32]. The linearization process is shown as follows:

(12) $$\bar{\phi_{i}}=\tilde{\phi_{i}}-ak_{i}-b=\phi_{i}-\frac{\phi_{n}-\phi_{1}}{k_{n}-k_{1}}k_{i}-\frac{1}{n}\sum_{j=1}^{n}\phi_{j},$$

where $\tilde{\phi }$ is the measured phase, $\phi_{i}$ is the actual phase, and $k_{i}$ is the index of the corresponding subcarrier.

#### 5.2.2. Abnormality Processing

Abnormal data have a great impact on training and testing performances. To remove the abnormal data points, we use the Pauta criterion, which is shown in the following equation:

(13) $$V_{i}^{F}=\left\{\begin{array}{cc} mean_{F} & |V_{i}^{n}-mean_{F}|>3std_{F},\\ 0, & else, \end{array}\right.$$

where, for the ith sample and Fth subcarrier, *V* is the CSI value, mean is the mean value and std is the standard deviation.

#### 5.2.3. Data Smoothing

**Figure 6:**
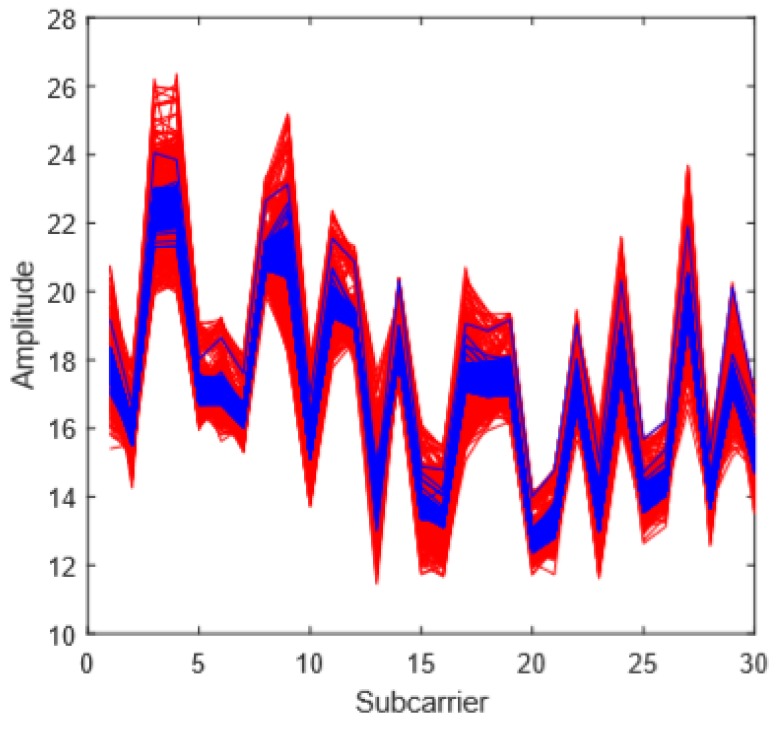
The data smoothing results.

Even after removing the outliers, there are still significant short-term fluctuations among adjacent samples. To remove the unwanted noise fluctuations, we use the moving average filter to process the data [33]. The method is shown in the following equation:

(14) $$\bar{CSI_{n}^{i}}=\frac{1}{m}\left(CSI_{n}^{i}+CSI_{n-1}^{i}+\ldots +CSI_{n-m+1}^{i}\right),$$

where *m* is the size of the sliding window, *n* is the index of the data packets, and *i* is the index of the subcarriers. There is a trade-off between sliding window size, i.e., data smoothness, and signal integrity. Figure 6 shows an example of data smoothing. The red lines are the original data, while the blue lines are the smoothed one. The data fluctuations are significantly suppressed. After data smoothing, the data are classified using machine learning techniques.

### 5.3. Result Analysis

In this section, we evaluate the performance of our techniques.

#### 5.3.1. Performance Comparison

In this work, we compare the performance of the following techniques:

1. Confidence: the state-of-the-art CSI based localization method [20], which uses the mean and standard deviation features extracted from CSI amplitude.
2. Statistics: a technique similar to the confidence method but employs four amplitude features and four phase features.
3. VG: a localization technique using only VG network features.
4. Combined: our technique which utilizes both VG and statistics features.

**Figure 7:**
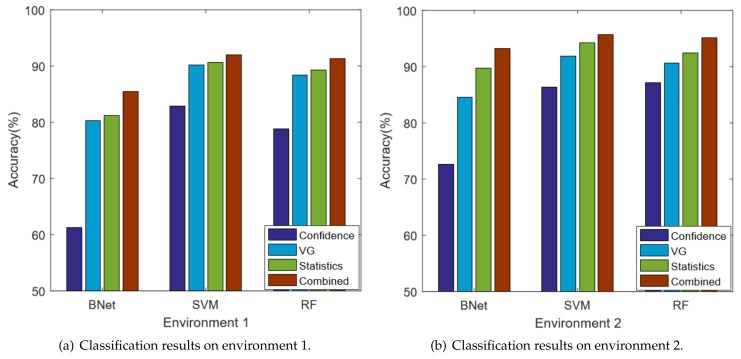
The comparison results.

We compare the classification accuracy at two different environments and the results are shown in Figure 7. In the figure, the *x*-axis is the three machine learning based classification algorithms and the *y*-axis is the classification accuracy. Among the four compared algorithms, both VG and Combine are our proposed techniques. In both environments, the Statistics method outperforms the Confidence method for all of the three classification methods. This implies that using only the mean and standard deviation features is insufficient to capture the CSI signal spatial variations. Moreover, the combined method constantly gives the best performance, which demonstrates that the network features are truly correlated to the CSI spatial variations. In other words, different indoor locations have an impact on the adjacent subcarrier correlations.

In general, by using the SVM technique, we can achieve the best performance in both environments. For the noisy environment 1, the Confidence method has 80.1% accuracy; the VG only method is 87.2%; the Statistical method is 91.7%; and the Combined method can achieve 92.0% accuracy. Our technique is significantly better in noisy environments, which has 11.9% improvement compared with the state-of-the-art Confidence method. However, the performance improvement is less significant in environment 2. For the best SVM algorithm, our combined method achieves 95.7% accuracy, which has about 8.9% improvement compared with the Confidence method, but only 2.8% compared with the Statistics method. It is possible that the VG network features and statistics features overlap with each other and cause over-fitting.

#### 5.3.2. Performance Analysis

**Figure 8:**
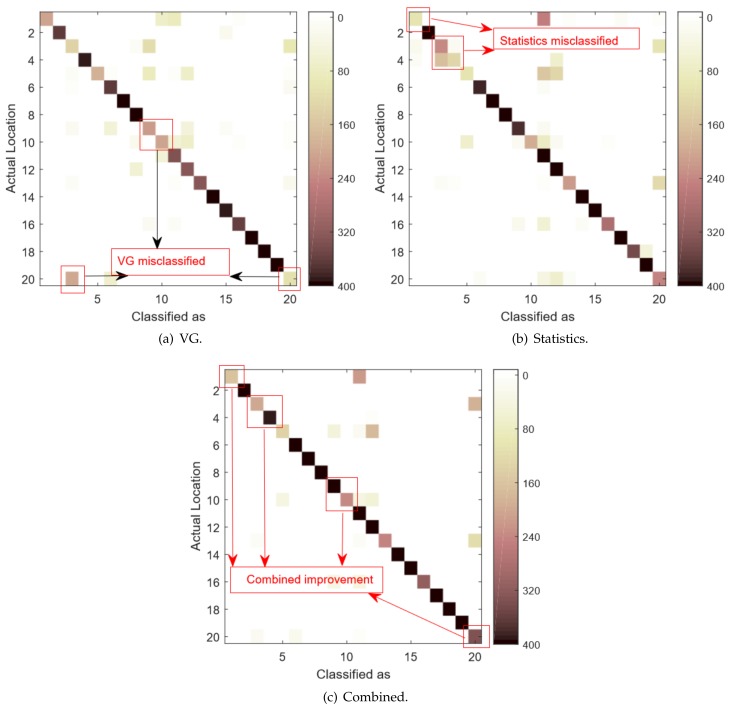
The confusion matrices of different methods.

To better illustrate and explain the performance of our combined technique, we plot the confusion matrices of the VG, Statistics, and Combined methods under the complex environment 1, as shown in Figure 8. In the figure, the *y*-axis is the indices of actual locations and the *x*-axis is the classification result frequencies. The orthogonal line represents the correctly classified results and the color intensities represent the frequencies.

Figure 8a,b show the corresponding confusion matrices of VG and Statistics methods, respectively. As illustrated in the figure, the misclassification locations for the VG and Statistics methods are mostly different. The annotated areas in Figure 8a,b show the corresponding misclassified locations. They are in different diagonal positions, while implying that, whenever one technique misclassifies, the other one may derive the correct result. Therefore, by combining the two different classes of features, the classification results of the combined technique can be significantly improved. For example, Figure 8a shows that location 20 is frequently misclassified as locations 3 and 6. Figure 8b shows that location 20 is frequently misclassified as locations 6, 11, 12, and 13. However, as shown in Figure 8c, by combining the features, location 20 is rarely misclassified except for few cases. It is clear evidence that the network features, which represent the inter-subcarrier correlations, are different from the statistics features generalized from individual subcarrier and hence can help improve the localization performance.

It should be noted that, although our technique improves significantly, there are still some misclassifications. The reason is as follows.

1. The CSI signatures contain significant noises. Because of environment variations and multi-path effects, the CSI signals are usually unpredictable and fluctuating, which can greatly affect the localization accuracy. For example, in many scenarios, we observe that the CSI signatures of a person standing in certain locations in the middle are very close to the signatures where one stands in the corner. In that case, those two locations are indistinguishable for any CSI based techniques. Therefore, there are always possibilities for misclassifications.
2. The feature selection methods can also cause misclassifications. In this work, we do not use raw CSI data directly. Instead, we extract features from them. Features can filter out the noises and simplify the calculation. However, it is also possible to omit useful information. Our technique is based on two feature sets, which are Statistical and VG features. They stand for the intra and inter correlations of subcarriers, respectively. It is demonstrated in Figure 7 that both feature sets can cause misclassifications. Therefore, for the locations where both feature sets predict incorrectly simultaneously, our technique also misclassifies.

**Figure 9:**
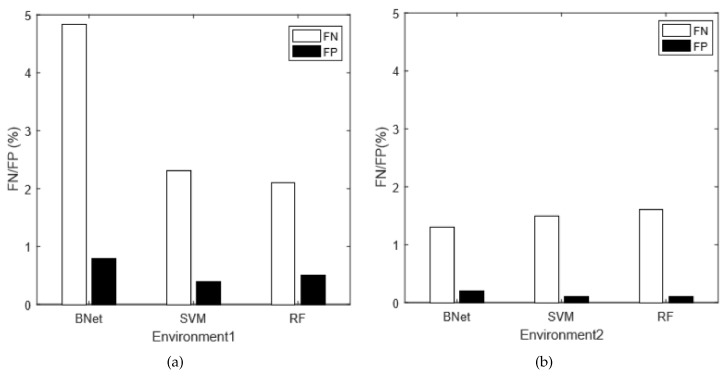
The FN/FP ratios on different environments.

To further analyze the classification results, we compare the false negative (FN) rate and false positive (FP) rate of our combined technique under different environments. Figure 9 shows the comparison results. For the best machine learning algorithm (SVM), the FN rates are constantly below 3% and the FP rates are below 1%. The performances of our algorithms are outstanding. Moreover, for both environments, the FP rate is significantly lower than the FN rate. It is a desirable result since, in most applications, the FP rate is much more important than the FN rate.

#### 5.3.3. Parameter Selection

**Figure 10:**
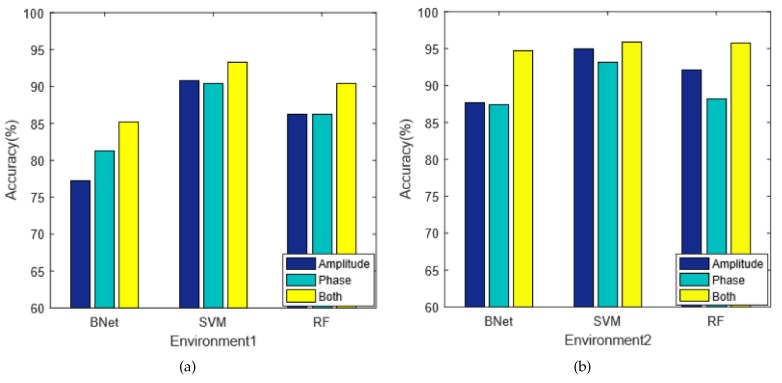
The amplitude/phase selection on classification accuracy.

We first discuss the selection of amplitude and phase parameter of the CSI signal. We compare the performance of our Combined technique using three different types of CSI data, which are amplitude, phase, and both, respectively. The comparison results are shown in Figure 10, where the *x*-axis is the three machine learning algorithms and the *y*-axis is the classification accuracy. The figure demonstrates that, by combining the amplitude and phase information, we can observe a universal improvement. The reason is that the amplitude and phase information represents different aspects of CSI characteristics.

**Figure 11:**
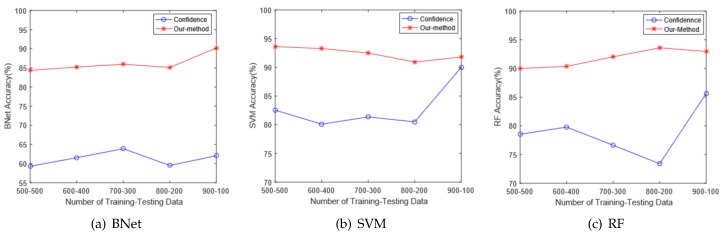
The impact of training and testing sizes.

Then, we discuss the impact of training and testing set sizes on algorithm performances. Figure 11 shows the evaluation results at the noisy testing environment 1. Our technique is significantly better than the Confidence method. As the size of the training set grows, the performances improve slightly. Moreover, it is possible that the machine learning based classifiers become overfitted. In that case, the classification performance may even drop slightly, as shown in Figure 11b. Considering the trade-off between training time and classifier performance, we employ 6:4 as the ratio to split the training and testing sets.

Table 1 shows the classification accuracy and the corresponding training time. The SVM method has the best performance and moderate training time. Meanwhile, the Bayes net approach requires the least training time. Therefore, there is a trade-off between algorithm performance and the time taken to obtain each model. The specific machine learning technique should be determined by considering all the requirements.

**Figure 12:**
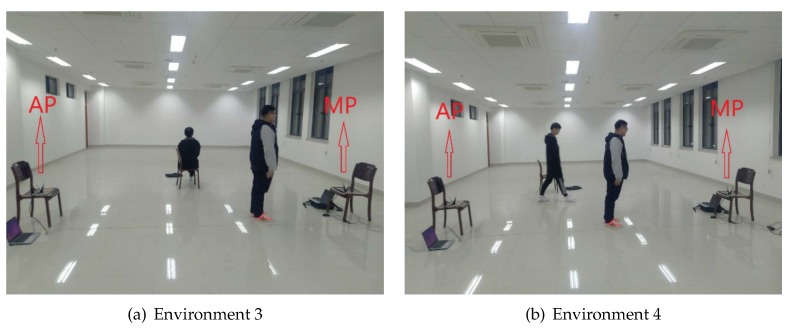
The impact of human interferences.

**Figure 13:**
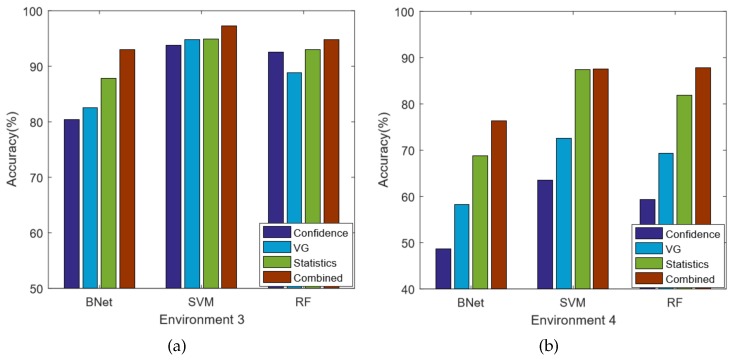
The impact of human interferences.

**Figure 14:**
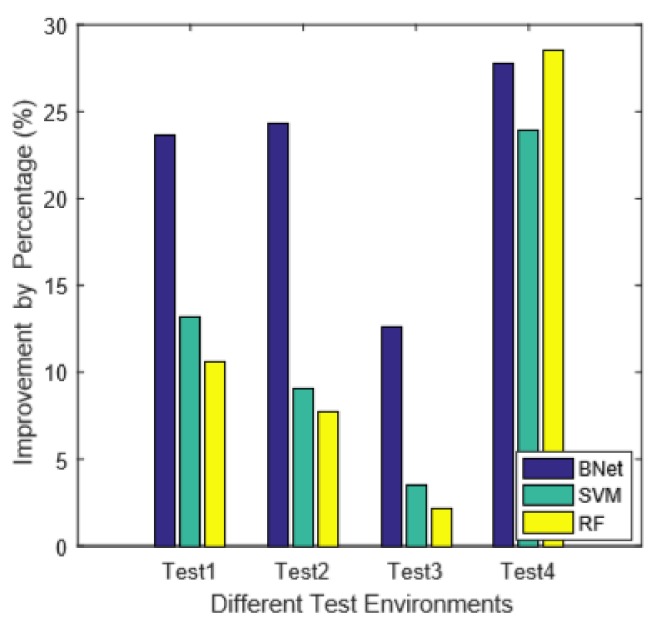
The overall improvements compared with the state-of-the-art technique.

Finally, we evaluate the influences of noise disturbance, e.g., presence of other persons, on algorithm performances. We add two more testing environments, which are environments 3 and 4, respectively. Figure 12 shows the details of these two environments. On testing environment 3, besides the testing personal, an additional person sits idly nearby. On testing environment 4, an additional person walks randomly inside the room. Figure 13 presents the corresponding experimental results. The results show that our technique is very robust compared with the existing methods. Especially in environment 4, the Confidence method is affected significantly by the walking person. Even for the SVM method, the classification accuracy drops below 65%, while, for our combined methods, the accuracy is still about 90%. Therefore, the experimental results demonstrate that the idly seated person does not have a significant impact on CSI based classification techniques. The human activity, on the other hand, can have significant impact. However, by considering both intra-subcarrier statistical features and inter-subcarrier network features, our Combined technique shows great resilience for such disturbance. Figure 14 shows the performance improvements compared with the Confidence method. The noisiest testing environment 4 records the greatest improvements. In general, there is a trade-off between noises and classification accuracy. However, by considering both intra and inter subcarrier correlations, our algorithm is demonstrated to be very noise resilient.

## 6. Conclusions

In this work, we propose a CSI based indoor localization technique which utilizes both the intra-subcarrier statistics and inter-subcarrier network features. We employ the VG method to transform the CSI amplitude and phase information into complex networks and extract the network features accordingly. Then, we combine the network features with traditional statistics features and use three widely use machine learning algorithms to determine the specific indoor locations. The experimental results show that our technique can achieve 96% classification accuracy, which is about a 9% improvement compared with the state-of-the-art CSI method. Moreover, it is demonstrated that our techniques are more robust to the environment noises and disturbances.

## Figures and Tables

**Table 1:** Performance and training time for machine learning algorithms.

Environment	BNet		SVM		RF	
	Acc(%)	Time(s)	Acc(%)	Time(s)	Acc(%)	Time(s)
Env1	85.21	0.56	93.28	1.79	90.70	4.92
Env2	84.74	1.15	95.90	3.21	95.74	8.84
